# Ultra-processed food consumption and metabolic syndrome: a cross-sectional study in Quilombola communities of Alagoas, Brazil

**DOI:** 10.1186/s12939-022-01816-z

**Published:** 2023-01-17

**Authors:** Lídia Bezerra Barbosa, Nancy Borges Rodrigues Vasconcelos, Ewerton Amorim dos Santos, Tamara Rodrigues dos Santos, Thays Ataide-Silva, Haroldo da Silva Ferreira

**Affiliations:** 1 Postgraduate Program in Health Sciences of the Institute of Biological and Health Sciences, Federal University of Alagoas, Campus A. C. Simões, BR 104, km 14, Tabuleiro dos Martins, Maceió, Alagoas 57072-970 Brazil; 2 Faculty of Nutrition of the Federal University of Alagoas, Campus A. C. Simões, BR 104, km 14, Tabuleiro dos Martins, Maceió, Alagoas 57072-970. Brazil; 3 Faculty of Medicine of the Federal University of Alagoas, Campus A. C. Simões, BR 104, km 14, Tabuleiro dos Martins, Maceió, Alagoas 57072-970. Brazil

**Keywords:** Metabolic syndrome, Eating, Industrialized foods, Fast foods, Ultra-processed food, Síndrome metabólica, Ingestão de Alimentos, Alimentos Industrializados, Fast Foods, Alimentos ultraprocessados

## Abstract

**Background:**

The processing of food can cause changes that turn them into risk factors for chronic diseases. A higher degree of food processing is associated with the development of chronic non-communicable diseases (NCDs), including the metabolic syndrome (MetS). The objective of this study was to analyze the relationship between ultra-processed food (UPF) consumption and the prevalence of MetS and its risk factors, focusing on a population group especially subjected to precarious living conditions and food insecurity.

**Method:**

Cross-sectional population-based study with women (19 to 59 years) from Quilombola communities of Alagoas. The socioeconomic, demographic, anthropometric, health status, lifestyle, and food intake (24-h recall) variables were analyzed. The dependent variable was the MetS, defined using the harmonization criteria of the Joint Interim Statement, and its components. The foods consumed were categorized according to the Nova Classification, assuming the highest UPF consumption as risk exposure. The measure of association was the prevalence ratio (PR) and respective 95%CI, calculated by Poisson regression with robust variance. We also analyzed the association with the Nova score of UPF consumption.

**Results:**

We investigated 895 women (38.9 ± 11.0 years), of whom 48.3% had MetS. On average, 15.9% of the total energy intake came from UPF. Lower Nova scores were associated with a lower prevalence of diabetes and low HDL. Higher UPF consumption was associated with a 30% higher prevalence of hypertension (PR = 1.30; 95%CI: 1.06–1.61).

**Conclusion:**

The highest UPF consumption was positively associated with the prevalence of hypertension, while a lower Nova score was a protective factor against diabetes and low HDL. UPF consumption in Quilombola communities is important but lower than that observed in the Brazilian population in general. It is suggested that public health programs be implemented to promote healthy eating while valuing the existing eating habits and traditions among the remaining Brazilian Quilombola people.

**Supplementary Information:**

The online version contains supplementary material available at 10.1186/s12939-022-01816-z.

## Resumo

**Introdução:**

O processamento de alimentos pode causar alterações que os transformam em fatores de risco para doenças crônicas. Um maior grau de processamento de alimentos está associado ao desenvolvimento de doenças crônicas não transmissíveis (DCNT), incluindo a síndrome metabólica (SM). O objetivo deste estudo foi analisar a relação entre o consumo de alimentos ultraprocessados (AUP) e a prevalência de SM e seus fatores de risco, com foco em um grupo populacional especialmente submetido a precárias condições de vida e insegurança alimentar.

**Método:**

Estudo transversal de base populacional com mulheres (19 a 59 anos) de comunidades Quilombolas de Alagoas. Foram analisadas as variáveis socioeconômicas, demográficas, antropométricas, estado de saúde, estilo de vida e ingestão alimentar (recordatório de 24 horas). A variável dependente foi a SM, definida a partir dos critérios de harmonização do *Joint Interim Statement*, e seus componentes. Os alimentos consumidos foram categorizados de acordo com a Classificação Nova, assumindo como exposição de risco o maior consumo de AUP. A medida de associação foi a razão de prevalência (RP) e respectivo IC 95%, calculado por regressão de Poisson com variância robusta. Também analisou-se a associação com o Escore Nova de consumo de AUP.

**Resultados:**

Foram investigadas 895 mulheres (38,9 ± 11,0 anos), das quais 48,3% tinham SM. Em média, 15,9% da ingestão total de energia provinham de AUP. Escores Nova mais baixos foram associados a uma menor prevalência de diabetes e HDL baixo. O maior consumo de AUP se associou a uma prevalência 30% maior de hipertensão arterial (RP = 1,30; IC95%: 1,06-1,61).

**Conclusão:**

O maior consumo de AUP se associou positivamente à prevalência de hipertensão, enquanto o menor escore Nova foi fator de proteção contra diabetes e baixo HDL. O consumo de AUP nas comunidades Quilombolas é relevante, mas inferior ao observado na população brasileira em geral. Sugere-se a implementação de programas de saúde pública que promovam a alimentação saudável, valorizando os hábitos e tradições alimentares existentes entre os remanescentes Quilombolas brasileiros.

## Introduction

Unhealthy eating patterns influence the occurrence of several chronic non-communicable diseases (NCDs), including metabolic syndrome (MetS), characterized by the presence of a set of cardiovascular risk factors in the individual [1–3]. The prevalence of MetS in adults ranges from 11.0 to 62.5% in different geographic regions of the world [4]. Some studies indicate that the prevalence of MetS is influenced by the ethnic-racial factor, with high prevalences being found in Afro-descendant populations. In Brazil, the prevalence of MetS ranged from 25.8% [5] to 55,4% [6] among African descendants living in Quilombola communities.

The main risk factors for developing MetS are use alcohol abuse, smoking, a sedentary lifestyle, and unhealthy eating habits [7–9]. Changes in the eating habits of the population have contributed to the occurrence of MetS due to the increased consumption of foods subjected to a high level of industrial processing [10], with consequent alteration of their natural state, to make them less perishable and improve their organoleptic characteristics, such as color, aroma, and flavor [11, 12].

Studies have shown that a higher degree of food processing is associated with the development of NCDs, constituting a risk factor for hypertension [13], obesity [14, 15], cancer [16], and MetS [17]. This fact is attributed to the high content of sodium, refined carbohydrates, saturated fats, and *trans* fats often present in these foods. These constituents act synergistically, increasing the risk of several morbid conditions and the etiology of MetS [13, 18, 19].

Currently, there are different classifications regarding the level of food processing. One of them is the so-called Nova classification [20], which has been incorporated into the guidelines in the Food Guide for the Brazilian Population [21]. This classification establishes four categories of foods according to the processing degree: 1) unprocessed and minimally processed foods; 2) processed culinary ingredients; 3) processed foods, and 4) ultra-processed foods (UPF). Among these categories, UPF are defined as a group of foods that include industrial food formulations made mostly or entirely from food-derived or laboratory- synthesized substances [22, 23].

The consumption of UPF is present in the food of individuals from all income levels, jeopardizing potentially the food security situation of different populations [24, 25]. Thus, populations living in a situation of social vulnerability, as in the case of the remaining Quilombola communities, need to have their food consumption better known since an unhealthy dietary pattern puts the health of individuals health at risk [24]. However, there are few studies on the relationship between food consumption of different processing levels and MetS [26], mainly among the Quilombola people.

Research conducted with socially vulnerable populations, characterized by difficulty of access to health services and adequate food, can contribute to the better clinical practice of health professionals and the planning of effective public policies to promote the quality of life of this population contingent.

This research aimed to characterize UPF consumption and identify its association with MetS and its risk factors in women from Quilombola communities in the state of Alagoas, Brazil.

## Methods

This study is part of a project entitled "Diagnosis of Health and Food and Nutritional Security of the Families of the Remaining Quilombos Communities in the State of Alagoas", which was approved by the Research Ethics Committee of the Federal University of Alagoas (CAAE 33.527.214.9.0000.5013). All women analyzed agreed to participate in the study and signed the Informed Consent Form.

### Study design and location

This is a population-based cross-sectional study, conducted in Quilombola communities of Alagoas, which, in 2015, comprised 68 communities certified as remaining quilombos, distributed in 34 of the state's 102 municipalities [27]. Alagoas has an estimated population of 3,351,543 inhabitants and has the worst Human Development Index (HDI = 0.683) among the other states of the federation [28]. The per capita monthly income of Quilombola families in Alagoas in 2015 was R$ 77.00, mainly from government contributions (social programs and pensions) and family farming [27, 29].

### Population, eligibility criteria, and sample design

The target population for this study was the women from Quilombola communities in Alagoas, aged 19 to 59 years. To obtain the sample size planned for the larger project of which this study is part, 34 of the 68 Quilombola communities in Alagoas state were selected by systematic sampling. The sampling process provided for obtaining a probabilistic sample representative of the families of Quilombola communities in the state [30].

The following eligibility criteria were established for this study: residing in one of the selected quilombola communities and belonging to the target age group. Exclusion criteria were being pregnant or lactating and having consumed alcohol in the last 24 h. Data from women who did not have information to define MetS, as well as those who reported implausible energy intakes (< 400 or > 4800 kcal/day) [31], were excluded from the analysis, given that such values are associated with under- or hyper-reports [32].

With these criteria, a sample of 895 women was obtained. Therefore, the sampling error was calculated a posteriori, considering the following parameters: MetS prevalence of 28.8% [5]; a universe of 6,465 women (estimating the existence of 6,465 Quilombola families in 2015 in Alagoas and assuming that in each household there would be one woman in the target age group); 1.2 for correction of the effect of complex sampling design; and a 95% confidence interval. After the calculations performed in the StatCalc application of the Epi Info 7.2 software (CDC, Atlanta, USA), the sampling error for investigating MetS in this sample was 3.1%.

### Data collect

Data collect occurred between April 2017 and January 2018 through interviews conducted during home visits, using structured forms tested in a pilot study. Interviewers were trained to standardize procedures and minimize errors in applying the forms. Demographic, socioeconomic, lifestyle, health, and food consumption variables were collected. All the data were collected at a single opportunity. During the interviews, blood pressure was measured and anthropometric data were collected. The biochemical tests were performed in a predetermined place in each community, to which the women were referred.

The economic class was defined based on the Criterion for Economic Classification Brazil (CCEB), adopted by the Brazilian Association of Research Companies [32], which distinguishes families in classes A, B1, B2, C1, C2, and D + E. This classification is established based on the possession of consumer goods at home, the education of the head of the family, and the infrastructure conditions of the residence. According to a scoring scale, families are classified in descending order, with class D + E being the lowest and class A representing the stratum with the highest economic status [33].

Blood pressure was measured in duplicate with the individual seated after 15 min of rest using Omron® digital devices, model HEM-7200. A third measurement was performed if there was a difference of more than 20 mmHg between the two measurements. A minimum interval of five minutes was established between measurements. For analysis, the most discrepant value was disregarded and the average of the valid values was considered.

The anthropometric variables assessed were body mass index (BMI), neck circumference (NC), waist circumference (WC), and waist-to-height ratio (WHtR). BMI was obtained from weight and height data (kg/m2). The weight was measured using a Seca® model 813 digital scale, with a capacity of up to 200 kg and sensitivity of 100 g, and height was obtained with the use of a Seca® portable stadiometer, model 213, able to measure up to 205 cm. WC and NC were measured with a 150 cm inextensible tape measure and sensitivity to 0.1 cm.

Biochemical tests were performed without prior fasting [34] to define the blood levels of glycated hemoglobin (HbA1C), triglycerides (TG), and high-density lipoprotein cholesterol (HDL). Blood drops obtained by digital puncture with disposable lancets were used. HbA1C was determined using the *Alere NycoCard Reader II®* (Abbott, USA) device, and HDL and TG were determined using the *Alere Cholestech LDX System®*.

### Variables

The dependents variables were the MetS (defined according to the Joint Interim Statement—JIS harmonization criteria, with adaptation) [35] and its components: (a) Abdominal obesity: WC ≥ 80 cm; (b) Hypertriglyceridemia: TG ≥ 175 mg/dL; (c) Low HDL: HDL < 50 mg/dL; Hypertension: systolic blood pressure ≥ 130 or diastolic blood pressure ≥ 85 mmHg; (e) Diabetes mellitus HbA1C ≥ 6.5 mg/dL, or being under drug treatment for hypertriglyceridemia, hypertension or diabetes. The adaptation consisted of replacing fasting blood glucose, as recommended by the JIS, by using HbA1C as a criterion for the classification of glucose homeostasis, a procedure recommended by the Brazilian Diabetes Society [36]. MetS was defined by the presence of at least three components among those specified in the JIS.

The independent variable was the highest consumption of UPF. We used the 24-h dietary recall (R24h), a method consisting of identifying, through an interview, all the food, and beverages ingested in the previous 24 h. A photographic record album was used to reduce memory bias and to facilitate the quantification of food portions [37]. A single R24h was applied to the study sample.

The quantities of food consumed reported in home measures were converted into grams and milliliters using home measure tables [37–40]. In the analysis of food nutritional composition, the food composition table of the U.S. Department of Agriculture was used as a base [41]. National reference tables were used for foods belonging to the Brazilian food culture [42–44]. According to the Nova classification, consumed foods were divided into four food groups [22]: 1) fresh and minimally processed foods; 2) processed culinary ingredients; 3) processed foods, and 4) UPF. Due to the objective proposed for this study and to have greater clarity in the results, only the ultra-processed group was analyzed as risk exposure, with the lowest consumption used for comparison.

The percentage contribution of the daily energy intake (%/day) of each food group according to the processing degree was obtained by calculating the energy value of each food group by multiplying the value obtained by 100 and dividing the result by the total energy intake of each woman. For the analyses, the percentage of contribution of daily energy intake of the UPF was used (%kcal/day).

In addition, we used the yes-or-no questions about food intake the previous day for 23 UPF subgroups from the Surveillance System of Risk and Protective Factors for Chronic Diseases by Telephone Survey (VIGITEL) questionnaire [45], which makes up the Nova score for UPF intake [46]. Each subgroup of UPF received a score of 0 (did not consume) or 1 (consumed). Since the list of foods in the Nova score is limited, other foods consumed by Quilombola women with characteristics similar to the foods belonging to the protocol originally proposed based on the results of the 2008/2009 Household Budget Survey (POF) were grouped into the subgroups of the list [47].

### Covariables

The covariates used to control for possible confounding factors and characterize the sample were:

- **demographic and socioeconomic variables:** age (19–29, 30–39, 40–49, and 50–59 years); self-reported skin color (black + brown and different from black + brown); education level in years of study (never studied, 1–4 years, 5–7 years, and ≥ 8 years); marital status (single, married, and widowed/divorced); economic class (A + B + C or D + E); participation in government social programs (yes or no); family income (≤ 1 minimum wage and > 1 minimum wage); employment status (employed or unemployed); food insecurity (yes or no), measured based on the Brazilian Scale of Food Insecurity (EBIA) [48].
- **Lifestyle and health-related variables:** alcoholism (yes or no); smoking (yes or no); physical activity level (PAL)—active or sedentary—obtained by applying the International Physical Activity Questionnaire (IPAQ), short version [49]; presence of health problems reported as having occurred in the last 15 days (yes or no).
- **Anthropometric variables:** Nutritional status was analyzed according to BMI (kg/m^2^), considering the overweight category**:** BMI ≥ 25 kg/m^2^ (yes or no) [50]. In addition, NC ≥ 34 cm (yes or no) [51] and WHtR ≥ 0.53 (yes or no) [52] were also analyzed and were considered predictors of cardiometabolic diseases.

### Data analysis

Data, except for food consumption data, were typed in a double independent entry in the Epi info version 3.5.3 software for data comparison and correction of possible typing errors. The food consumption data were entered in the software Dietpro® Clinico, version 6.0. After completing the first typing stage, all data were checked against the electronic spreadsheet obtained with the physical forms, and, when necessary, typing errors were corrected. After this, the consumption data were exported to Microsoft Excel® software.

Descriptive analyses were performed for all variables. Categorical variables were expressed as absolute and relative frequencies, while for continuous or discrete variables, medians, interquartile ranges, means, and standard deviations were used, according to the distribution normality, evaluated by the Kolmogorov–Smirnov test.

The values of the percentage contribution of daily energy intake according to the degree of food processing were classified according to quartiles into four categories. The 1^st^ quartile (Q1) was considered the lowest or no consumption; the 2^nd^ quartile (Q2) was considered moderate consumption; and the quartiles above the median (Q3 and Q4, respectively) were considered higher consumption. The consumption of UPF was evaluated by comparing Q2, Q3, and Q4 with Q1.

The Nova score of each participant's UPF consumption was calculated by adding the score values of each UPF subgroup consumed among the 23 listed, and this score could range from 0 to 23. In the analysis, the scores were categorized as follows: ≤ 1; 2; 3; ≥ 4. Due to the low frequency observed for values equal to or greater than 4, all of these (4 to 23) were grouped into the fourth category. This category indicates a high UPF intake.

The MetS was compared with the different covariates to observe statistical differences between the categories for which Pearson's χ2 test was used. The crude and adjusted prevalence ratios (PR) with respective 95% confidence intervals (95%CI) were established using Poisson regression with robust variance adjustment. The covariates that presented a significance level of up to 20% (*p* < 0.2) in the crude model were included in the multivariable analysis. Previously, however, to avoid the problem of multicollinearity, the variables (demographics, socioeconomics, lifestyle, health, and anthropometrics) with the highest correlation (*r* = 0.7) were identified. According to Pearson's correlation matrix, only the anthropometric variables BMI and WHtR (*r* = 0.73) presented this characteristic. Thus, only BMI (i.e., overweight—yes or no) was kept in the multivariable analysis.

For the multiple model, three blocks were organized: model 1 (adjusted for demographic and socioeconomic features); model 2 (variables from model 1 with a 5% significance level plus information related to lifestyle and health); and model 3 (variables from models 1 and 2 with a *p*-value < 0.05 plus anthropometric data). In each analysis level, there was, respectively, successive elimination of non-significant variables (backward stepwise). The final adjusted model was composed of all the variables remaining in model 3.

Statistical analysis was performed in Stata/SE version 12.1 software (StataCorp LP. College Station, TX, USA).

## Results

A total of 895 women (38.9 ± 11.0 years) were investigated. The majority (56.0%) belonged to the age group of 30 to 49 years, were black (90.8%), married (79.4%), belonged to the economic class D + E (94.4%), and had less than eight years of schooling (71.0%). Overweight was identified in 69.0% of women (Tables 1 and 2).

**Table 1:** Distribution of metabolic syndrome (MetS) according to demographic and socioeconomic characteristics of Quilombola women in Alagoas, Brazil, 2018

Variables	Total (*n* = 895) n (%)	MetS (*n* = 432) n (%)	Crude PR (95% CI)	p^a^
**Age group (years)**				
19 a 29	211 (23.6)	41 (19.4)	1	-
30 a 39	263 (29.4)	119 (45.3)	2.33 (1.72–3.16)	< 0.001*
40 a 49	238 (26.6)	142 (59.7)	3.07 (2.29–4.12)	< 0.001*
50 a 59	183 (20.4)	130 (71.0)	3.66 (2.74–4.89)	< 0.001*
**Race/skin color (self-reported)**				
Different from brown or black	82 (9.2)	45 (54.9)	1	-
Black or Brown	812 (90.8)	387 (47.7)	0.87 (0.70–1.07)	0.186*
**Marital status**				
Single	111 (12.4)	39 (35.1)	1	-
Married (lives with a partner)	711 (79.4)	357 (50.2)	1.43 (1.10–1.86)	0.008*
Widowed/divorced	73 (8.2)	36 (49.3)	1.40 (0.99–1.97)	0.053*
**Schooling (complete years of study)**				
≥ 8	259 (29.0)	79 (30.5)	1	-
Never studied	112 (12.6)	73 (65.2)	2.14 (1.70–2.69)	< 0.001*
1–4	364 (40.8)	211 (58.0)	1.90 (1.56–2.33)	< 0.001*
5–7	157 (17.6)	69 (43.9)	1.44 (1.12–1.86)	0.005*
**Economic class **^**b**^				
B + C	50 (5.6)	20 (40.0)	1	-
D + E	844 (94.4)	411(48.7)	1.22 (0.86–1.72)	0.266
**Family participation in a government program**				
No	236 (26.4)	139 (58.9)	1	-
Yes	659 (73.6)	293 (44.5)	0.75 (0.66–0.87)	< 0.001*
**Family income (in minimum wage) **^**c**^				
> 1	248 (34.6)	110 (44.3)	1	-
≤ 1	468 (65.4)	236 (50.4)	1.14 (0.96–1.34)	0.130*
**Employment status**				
Employed	507 (57.1)	257 (50.7)	1	-
Unemployed	381 (42.9)	172 (45.1)	0.89 (0.77–1.02)	0.105*
**Food and nutritional insecurity**				
No	515 (58.3)	237 (46.0)	1	-
Yes	369 (41.7)	187 (50.7)	1.10 (0.96–1.26)	0.169*

*PR* Prevalence ratios, *95%CI* 95% confidence intervals

^a^Pearson χ^2^ test

^b^According to the Criterion for Economic Classification Brazil ^32^. (There were no families in class A)

^c^Minimum wage at the time of the survey: R$937.00 (US$293.70)

^*^Variable selected to compose the multivariate analysis (*p* < 0.2)

**Table 2:** Distribution of metabolic syndrome (MetS) according to lifestyle, health, and anthropometric characteristics of women living in Quilombola communities in the state of Alagoas, Brazil, 2018

Variables	Total (*n* = 895) n (%)	MetS (*n* = 432) n (%)	Crude PR (95% CI)	p^a^
**Smoking**				
No	684 (77.1)	304 (44.4)	1	-
Yes	203 (22.9)	122 (60.1)	1.35 (1.17–1.55)	< 0.001*
**Alcoholism**				
No	578 (65.2)	286 (49.5)	1	-
Yes	309 (34.8)	140 (45.3)	0.91 (0.79–1.06)	0.242
**Physical activity level**				
Active	531 (59.7)	259 (48.8)	1	-
Sedentary	359 (40.3)	173 (48.2)	0.99 (0.86–1.13)	0.864
**Health problems in the last 15 days**				
No	589 (65.9)	274 (46.5)	1	-
Yes	305 (34.1)	158 (51.8)	1.11 (0.97–1.28)	0.128*
**Overweight (BMI ≥ 25 kg/m**^**2**^**)**				
No	271 (31.0)	43 (15.9)	1	-
Yes	602 (69.0)	382 (63.5)	3.99 (3.02–5.30)	< 0.001*
**Waist-to-height ratio**				
Normal	336 (38.1)	54 (16.0)	1	-
High (≥ 0,53)	547 (61.9)	373 (68.2)	4.24 (3.30–5.45)	< 0.001*
**Neck circumference**				
Normal	503 (57.0)	159 (31.6)	1	-
High (≥ 34 cm)	379 (43.0)	268 (70.7)	2.24 (1.94–2.58)	< 0.001*

*PR* Prevalence ratios, *IC95%* 95% confidence intervals

^a^Pearson χ^2^ test

^*^Variable selected to compose the multivariate analysis (*p* < 0.2)

The prevalence of MetS was 48.3%. As for the components of MetS, 68.2% had abdominal obesity, 45.0% had hypertension, 28.8% had diabetes, 33.1% had hypertriglyceridemia, and 74.9% had low HDL (Supplementary Table 1).

In the crude analysis, the variables associated with a higher prevalence of MetS were older age, married status, low education, participation in government assistance programs, smoking, being overweight, and high WHtR and WC (Tables 1 and 2). The average energy intake was 1,437.2 kcal (± 627.9 kcal), and of this total, the average percentage contribution from the UPF group was 15.9%. The distribution of energy intake by quartiles is shown in Supplemental Table 2, where it is observed that the mean percentage of contribution of the caloric intake from UPA in relation to the total caloric intake was 40.5%.

Supplemental Table 3 shows the consumption frequency for each of the 23 food subgroups of the Nova score. Five food subgroups were most consumed: packaged snacks (or chips) or saltine crackers (26.0%); margarine (20.2%); sweet biscuits with or without filling (16.5%); powdered drink mix (14.5%); and loaves of bread, like of the hot dogs or hamburgers buns or similar (13.6%).

**Table 3:** Prevalence ratio of metabolic syndrome distribution according to consumption quarters derived from ultra-processed foods or categories according to Nova score values. Study with quilombola women in the state of Alagoas, Brazil, 2018

Categories	Total (*n* = 895) n (%)	Metabolic syndrome		PR (95% CI)	p^a^
		**No (*n* = 463)** **n (%)**	**Yes (*n* = 432)** **n (%)**		
**Quartiles of % energy intake from ultra-processed foods**					
Q1	227 (25.4)	120 (52.9)	107 (47.1)	1	-
Q2	221 (24.7)	115 (52.0)	106 (48.0)	1.02 (0.83–1.24)	0.861
Q3	224 (25.0)	109 (48.7)	115 (51.3)	1.09 (0.90–1.31)	0.373
Q4	223 (24.9)	119 (53.4)	104 (46.6)	0.99 (0.81–1.20)	0.915
**Nova score categories for consumption of ultra-processed foods**					
≤ 1	569 (63.6)	293 (51.5)	276 (48.5)	1.00	-
2	192 (21.4)	96 (50.0)	96 (50.0)	1.03 (0.87–1.22)	0.719
3	78 (8.7)	40 (51.3)	38 (48.7)	1.01 (0.79–1.28)	0.972
≥ 4	56 (6.3)	34 (60.7)	22 (39.3)	0.81 (0.58–1.13)	0.220

*PR* Prevalence ratios, *95% CI* 95% confidence intervals

Q1 = 1^st^ quarter; Q2 = 2 ^nd^ quarter; Q3 = 3^rd^ quarter; Q4 = 4^th^ quarter

^a^Pearson χ^2^ test

The Nova score distribution ranged from 0 to 9, and scores ≤ 1 (63.6%) and 2 (21.4%) were more frequent. The percentage of women with a score ≥ 4 (high UPF intake) was 6.3% (Table 3).

The associations of UPF consumption, Nova score, MetS, and its components are described in Tables 3, 4, and 5 (for the adjusted analysis, the variables present in each model are shown in Supplementary Tables 4 and 5).

**Table 4:** Adjusted prevalence ratio (PR) for metabolic syndrome and its components, according to Nova score categories for consumption of ultra-processed foods: Study with Quilombola women in the state of Alagoas, Brazil, 2018

Nova score	Adjusted PR					
	**Model 1**		**Model 2**		**Model 3**	
	PR (95%CI)	p	PR (95%CI)	p	PR (95%CI)	p
Arterial hypertension						
≤ 1	Reference	-	Reference	-	Reference	-
2	1.02 (0.80–1.30)	0.855	1.03 (0.81–1.31)	0.820	1.01 (0.79–1.29)	0.930
3	1.24 (0.87–1.76)	0.234	1.21 (0.84–1.73)	0.304	1.22 (0.85–1.74)	0.274
≥ 4	0.95 (0.60–1.50)	0.818	0.95 (0.60–1.50)	0.826	0.89 (0.57–1.42)	0.640
Diabetes mellitus (HbA1C ≥ 6,5 mg/dL)						
≤ 1	Reference	-	Reference	-	Reference	-
2	0.97 (0.74–1.26)	0.820	0.99 (0.76–1.29)	0.917	0.92 (0.70–1.21)	0.5432
3	0.56 (0.31–0.98)	0.044	0.59 (0.33–1.03)	0.065	0.56 (0.31–0.99)	0.046
≥ 4	0.82 (0.49–1.44)	0.517	085 (0.49–1.46)	0.557	0.82 (0.47–1,43)	0.492
Abdominal obesity (waist circumference ≥ 80 cm)						
≤ 1	Reference	-	Reference	-	Reference	-
2	0.96 (0.86–1.07)	0.466	0.96 (0.86–1.07)	0.455	0.98 (0.91–1.05)	0.506
3	1.06 (0.91–1.23)	0.462	1.06 (0.91–1.24)	0.456	1.03 (0.93–1.14)	0.573
≥ 4	0,98(0.80–1.21)	0.868	0.98 (0.80–1.20)	0.852	0.93 (0.83–1.05)	0.233
HDL low (HDL < 50 mg/dL)						
≤ 1	Reference	-	Reference	-	Reference	-
2	0.91 (0.82–1.01)	0.068	0.91 (0.82–1.01)	0.067	0.90 (0.81–0.99)	0.046
3	1.01 (0.89–1.15)	0.875	1.02 (0.90–1.16)	0.689	1.01 (0.90–1.15)	0.880
≥ 4	1.01 (0.86–1.16)	0.968	1.01 (0.86–1.16)	0.972	0.99 (0.86–1.15)	0.942
Hypertriglyceridemia (Triglycerides ≥ 175 mg/dL)						
≤ 1	Reference	-	Reference	-	Reference	-
2	1.01 (0.81–1.25)	0.976	1.01 (0.81–1.26)	0.934	1.01 (0.82–1.25)	0.911
3	1.22 (0.89–1.67)	0.215	1.21 (0.88–1.67)	0.246	1.23 (0.90–1.69)	0.195
≥ 4	0.83 (0.53–1.31)	0.428	0.83 (0.53–1.31)	0.427	0.79 (0.50–1.26)	0.322
Metabolic syndrome						
≤ 1	Reference	-	Reference	-	Reference	-
2	0.97 (0.81–1.16)	0,744	0.97 (0.81–1.16)	0.741	1.01 (0.86–1.20)	0.881
3	1.16 (0.90–1.50)	0.244	1.20 (0.93–1.54)	0.161	1.21 (0.94–1.56)	0.131
≥ 4	0.97 (0.70–1.35)	0.858	0.97 (0.70–1.35)	0.860	0.90 (0.67–1,22)	0.507

*PR* Prevalence ratios, *95%CI* 95% confidence intervals

Model 1: Adjusted for demographic and socioeconomic characteristics whose in the crude analysis showed *p* ≤ 0.20 (age, race/skin color, marital status, schooling, family participation in a government program, family income, employment status and food insecurity—See Supplementary Table 4

Model 2: Adjusted for lifestyle and health variables whose in the crude analysis showed p ≤ 0.20 (smoking and health problems in the last 15 days) added to the variables of model 1 that showed *p* < 0.05 in the analysis for the aforementioned model (these variables were different for each outcome evaluated; only age was present in the analysis for all outcomes)—See Supplementary Table 4

Model 3: adjusted for anthropometric variables whose in the crude analysis showed *p* ≤ 0.20 (excess weight and neck circumference) plus the variables from model 1 that showed *p* < 0.05 in the analysis of the model 1 (these variables were different for each assessed outcome; only age was present in the analysis for all outcomes). Lifestyle and health variables were not part of this final model as they did not present *p* < 0.05 in the analysis of model 2—See Supplementary Table 4

**Table 5:** Adjusted prevalence ratio for the metabolic syndrome and its components, according to the quartiles of the percentage of energy consumption from ultra-processed foods percentage energy contribution from the consumption of ultra-processed foods in relation to total energy intake, by quilombola women in the state of Alagoas, Brazil, 2018

Desfechos	Quartiles of the percentage of energy consumption from ultra-processed foods				*p*-value
	**Q1**	**Q2**	**Q3**	**Q4**	
	**PR (95% CI)**	**PR (95% CI)**	**PR (95% CI)**	**PR (95% CI)**	
**Arterial hypertension**					
Model 1	1.00	**1.31 (1.06–1.62)**	1.21 (0.97–1.50)	1.16 (0.91–1.49)	**0.014**^**a**^
Model 2	1.00	**1.31 (1.06–1.62)**	1.20 (0.97–1.50)	1.16 (0.91–1.49)	**0.014**^**a**^
Model 3	1.00	**1.30 (1.06–1.61)**	1.20 (0.97–1.51)	1.17 (0.92–1.48)	**0.013**^**a**^
**Diabetes mellitus**					
Model 1	1.00	1.22 (0.93–1.60)	1.22 (0.91–1.62)	0.92 (0.66–1.28)	0.612^b^
Model 2	1.00	1.21 (0.92–1.60)	1.22 (0.92–1.63)	0.91 (0.66–1.27)	0.601^b^
Model 3	1.00	1.21 (0.92–1.58)	1.19 (0.90–1.59)	0.89 (0.64–1.24)	0.498^b^
**Abdominal obesity**					
Model 1	1.00	0.98 (0.87–1.11)	0.94 (0.83–1.07)	**1.13 (1.01–1.26)**	**0.038**^**a**^
Model 2	1.00	0.98 (0.87–1.11)	0.94 (0.83–1.07)	**1.13 (1.01–1.26)**	**0.038**^**a**^
Model 3	1.00	1.04 (0.95–1.13)	1.02 (0.95–1.10)	1.06 (0.98–1.15)	0.136^b^
**HDL low**					
Model 1	1.00	0.97 (0.87–1.07)	0.93 (0.83–1.03)	0.95 (0.86–1.06)	0.398^b^
Model 2	1.00	0.97 (0.88–1.08)	0.94 (0.84–1.04)	0.96 (0.86–1.06)	0.436^b^
Model 3	1.00	0.97 (0.88–1.07)	0.93 (0.83–1.03)	0.96 (0.87–1.07)	0.466^b^
**Hypertriglyceridemia**					
Model 1	1.00	0.93 (0.72–1.20)	1.09 (0.86–1.39)	0.98 (0.76–1.28)	0.919^b^
Model 2	1.00	0.93 (0.72–1.20)	1.09 (0.86–1.40)	0.96 (0.74–1.25)	0.766^b^
Model 3	1.00	0.93 (0.72–1.20)	1.13 (0.89–1.43)	0.98 (0.75–1.27)	0.865^b^
**MetS**					
Model 1	1.00	1.02 (0.83–1.24)	1.08 (0.88–1.31)	1.11 (0.89–1.36)	0.340^b^
Model 2	1.00	1.02 (0.83–1.24)	1.08 (0.89–1.32)	1.11 (0.90–1.36)	0.418^b^
Model 3	1.00	1.03 (0.86–1.24)	1.14 (0.95 -1.37)	1.09 (0.89–1.32)	0.391^b^

*PR* Prevalence ratio, *95%CI* 95% confidence intervals, *Q1* 1^st^ quarter, *Q2* 2 ^nd^ quarter, *Q3* 3^rd^ quarter, *Q4* 4^th^ quarter, *MetS* Metabolic syndrome

Model 1: Adjusted for demographic and socioeconomic characteristics whose in the crude analysis showed *p* ≤ 0.20 (age, race/skin color, marital status, schooling, family participation in a government program, family income, employment status and food insecurity—See Supplementary Table 5)

Model 2: Adjusted for lifestyle and health variables whose in the crude analysis showed *p* ≤ 0.20 (smoking and health problems in the last 15 days) added to the variables of model 1 that showed *p* < 0.05 in the analysis for the aforementioned model (these variables were different for each outcome evaluated; only age was present in the analysis for all outcomes)—See Supplementary Table 5

Model 3: Adjusted for anthropometric variables whose in the crude analysis showed *p* ≤ 0.20 (excess weight and neck circumference) plus the variables from model 1 that showed *p* < 0.05 in the analysis for the aforementioned model (these variables were different for each outcome evaluated; only age was present in the analysis for all outcomes). The lifestyle and health variables were not part of this final model as they did not present *p* < 0.05 in the analysis of model 2—See Supplementary Table 5

^a^Values reported only for the quartile in bold that presented association; ^b^values referring to the 4^th^ quartile of each model

MetS was not associated with any category of the Nova score. However, in the adjusted analysis of the MetS components, it was observed that a lower Nova score (score 3) reduced the prevalence of diabetes by 44% (Table 4): PR = 0.56; 95%CI: 0.31–0.99; *p* = 0.046. The lower score was also an independent protective factor for low HDL (PR = 0.90; 95%CI: 0.81–0.99; *p* = 0.046). Hypertension, abdominal obesity, and hypertriglyceridemia were not associated with any level of the Nova score.

No association with MetS was found related to the percentage energy contribution of UPF consumption (Tables 3 and 5). However, it was found for its components that UPF consumption at a moderate level (Q2) increased hypertension prevalence by 30% (PR = 1.30; 95%CI: 1.06–1.61; *p* = 0.013) compared to the lowest consumption (Q1). The other components were not associated with UPF consumption (Table 5).

## Discussion

Compared to the UPF consumption described in studies conducted in populations belonging to more urbanized settings, we can assume that this eating pattern is less established among Quilombola women. According to data from the Vigitel-2020 survey, the consumption frequency of five or more UPF groups (on the day before the interview) for the population aged > 18 years was 18.5% in all 27 Brazilian capitals, and this frequency was 16.1% for women aged > 18 [53], i.e., approximately 2.5 times higher than for the Quilombola women (considering for these the consumption of 4 or more UPF groups).

It should be noted that the Quilombola women analyzed belong to rural communities that, due to cultural issues, often preserve the eating habits acquired from their ancestors who survived on subsistence agriculture and, thus, had a diet consisting basically of natural foods [54]. Today, however, in addition to these foods, the food practices reported revealing the inclusion of processed and ultra-processed foods in their food culture. This may be related to the difficulties faced in developing agricultural practices and the urbanization of cities with the construction of roads that lead more easily to urban areas [55]. In addition, the younger Quilombola generations have lost their relationship with the land and agriculture; therefore, their access to food has been influenced by the globalization process [56].

Regarding the food consumption of populations that have similarities with that of this study (social vulnerability), research comparing food patterns of white and black Americans found a higher frequency of unhealthy eating patterns (consumption of foods such as sausages, fried foods, refined grains, sugar, margarine, sweets, and fats) among blacks [57]. This observation is similar to what occurs in the Brazilian scenario, as shown by data from the National Health Survey, which show that black individuals and those with low socioeconomic status have a worse dietary profile [58]. In Brazil, this situation is a reflection of the precarious living conditions historically imposed on blacks, being a perverse legacy of the slavery process. As in other countries, in Brazil, the black population has a worse socioeconomic condition compared to whites [58].

Given the above, it is worrying how much UPF is available for commercialization in Quilombola communities. Regarding this issue, Serafim et al. [59], in a study carried out in a city in Brazil, found a higher score of availability of UPF in regions with lower income, a higher percentage of non-white people, and a higher number of residents per household. This finding demonstrates that vulnerable populations, such as the Quilombolas, are having their food consumption influenced by the industry and trade of unhealthy food products, especially UPF, which represents a risk to the health of the population due to its relationship with the development of NCDs [60].

The results regarding the Nova score are the first related to the analysis of its association with chronic diseases. A study with this instrument was conducted with the adult population of the 27 Brazilian state capitals using data from the Vigitel-2019 survey. This study showed that being male, younger, and having a lower level of education were factors associated with higher UPF consumption [61].

This study showed that, although no association was found between MetS and UPF consumption, higher consumption of these foods was related to higher hypertension prevalence. A lower Nova score was also a protective factor against diabetes and low HDL.

However, further studies with the female Quilombola population may be necessary since, due to the cultural and socioeconomic characteristics of Quilombola peoples, there was a low frequency of the higher categories of the Nova score, reducing the statistical power to demonstrate the associations investigated (the percentage of women with a score ≥ 5, previously established in the study by Costa et al. [46], was only 1.7%, considering that the score ranges from 0 to 23). It should be noted that in the present study, a score ≥ 4 was considered a high frequency of consumption.

The result that there was no association between the consumption of ultra-processed foods and MetS, unlike that observed in other studies [17, 19, 62], was similar to that found in a study of Lebanese adults, in which no relationship was found between the “ultra-processed food pattern” and MetS [26]. It is worth noting that although UPF consumption was not associated with MetS, it contributes a considerable percentage of women's energy intake, possibly suggesting a trend to transition from a healthy eating pattern to one of relatively high consumption of UPF pattern.

This study showed that even moderate consumption (Q2) of UPF was associated with hypertension. This result corroborates with the findings of a cohort study conducted in Brazil, where individuals with UPF consumption in the upper fifth of daily energy intake had a higher incidence of this pathology (PR = 1.35; 95%CI: 1.10–11.81) [13].

In our study, in the crude analysis up to level 2 of the multiple analysis, high UPF consumption was observed to be a risk factor for abdominal obesity. However, this association lost significance in the final adjusted model (level 3). A similar fact occurred in a study with young Brazilian adults, where UPF intake was found to be a risk factor for abdominal obesity only in the unadjusted analysis (OR = 1.09; 95% CI: 1.01–1.18) [63]. It is worth noting that level 3 was formed by the anthropometric variables, which present a strong positive correlation with the outcomes analyzed. Obviously, the power of these associations weakens the previous levels' relationships, which cannot always be avoided during the adjustment process because of residual effects that may remain.

Even considering the differences between the populations studied and the results achieved, UPF consumption should be cautiously adopted given the high proportion of artificial ingredients in their formulations [21], which are potentially harmful to health, especially when consumed in excess. There is evidence that excessive consumption of these foods is related to an increase in NCDs, such as obesity, cardiovascular, and metabolic diseases [18, 64–66].

This relationship is due to the poor nutritional quality of these foods, most of which are characterized by high saturated fat, *trans*-fatty acids, refined sugars, little or no fiber, vitamins, and minerals [67, 68].

Regarding abdominal obesity, which plays a central role in the triggering of several NCDs, one of the hypotheses supporting its origin related to UPF consumption concerns the high caloric density of these foods that can delay satiety signals and, as a consequence, larger portions are consumed [68, 69].

It is worth mentioning that this study evaluated African-descent women living in Quilombola communities and subjected to great social vulnerability, institutional racism, and food insecurity [30, 70, 71]. Studies on Quilombola communities are still limited, being this the first regarding the consumption of UPF. Because of these characteristics, this population requires priority attention from the government to correct historical distortions and promote health and quality of life. Such aspects justify the importance of studies that can support these actions' planning and implementation.

In this aspect, the importance of food and nutrition education activities stands out. The encouragement and strengthening of healthy eating habits with increased consumption of fresh foods are important practices for preventing MetS and other NCDs [13, 72].

There are few studies that, using the Nova classification, evaluated the relationship between the level of food processing and MetS. Proof of this can be seen in the fact that in a systematic review conducted by Santos et al. [73], only two studies addressing this issue were included. In addition to the studies included in the aforementioned review, later publications by Martinez-Perez et al. [74] and Phillips et al. [75] also investigated the association between UPF and cardiometabolic markers. However, none of them used the Nova score and its association with MetS.

The cross-sectional design is a limitation of this study because of the possible temporality bias. Another limitation concerns the application of a single R24h. Thus, the food consumption data cannot reflect the usual consumption of the women assessed. However, it is noteworthy that several other studies also used a single R24h to assess food intake [26, 52, 76]. In addition, the inclusion of only women in the sample of the present study can be considered another limiting factor. However, it is noteworthy that women influence the composition of family food [77], being considered as the main ones responsible for promoting the food security of their families [78]. The non-inclusion of males in our survey was defined due to two situations: (a) Household surveys are carried out on different days of the week and, almost always, men are not at home, but at their workplaces; (b) In a much higher proportion than other family members, men refuse to participate in the study.

The strengths of the work include the unprecedented nature of the study and the use of a representative sample of a specific population recognized as a priority in the research agenda of the Ministry of Health [79]. It is the first to use the Nova score of UPF consumption in a Quilombola population. Calibrated instruments, anthropometric techniques, and standardized laboratory tests were adopted for the suitable definition of exposure and outcome. Confounding factors were controlled for using hierarchical multiple analysis. We used the Nova classification (in addition to the Nova score of UPF consumption), which is appropriate and recommended worldwide for developing research and policies on food, nutrition, and public health [80–82]. With all these characteristics, the study presents internal and external validity, so that the results can be extrapolated to Quilombola populations in other Brazilian states.

It is worth pointing out that the results presented are relevant for a greater understanding of the Quilombola population's eating habits and their relationship to health, despite being carried out only with women, and may represent the practices of other residents, since women, who are mainly responsible for managing the food in the home, play a key role in shaping the family's eating habits.

## Conclusion

The highest UPF consumption was positively associated with the prevalence of hypertension, while a lower Nova score was a protective factor against diabetes and low HDL.

UPF consumption in quilombola communities is important but still lower than that observed in the Brazilian population in general.

It is suggested that public health programs be implemented to promote healthy eating while valuing the existing eating habits and traditions among the remaining Brazilian Quilombola people.

## Supplementary Information

**Additional file 1: Supplementary Table 1.** Distribution of the metabolic syndrome according to the categories of its components in quilombola women from Alagoas, Brazil, 2018. **Supplementary Table 2.** Mean total energy intake and absolute and relative contribution of this intake that was derived from ultra-processed foods. **Supplementary Table 3.** Percent distribution of the consumption, on the day before the interview, of food groups included in the Nova score of consumption of ultra-processed foods by Quilombola women in Alagoas, Brazil, 2018. **Supplementary Tables 4.** Description of the covariates (confounding factors) controlled in the adjusted analysis of table 4, according to the outcome evaluated. **Supplementary Tables 5.** Description of the covariates (confounding factors) controlled in the adjusted analysis of Table 5, according to the outcome evaluated.

## Data Availability

The datasets used and/or analysed during the current study are available from the corresponding author upon reasonable request.

## Abbreviations

95% CI: 95% Confidence intervals
BMI: Body Mass Index
CECB: Criterion for Economic Classification Brazil
EBIA: Brazilian Scale of Food Insecurity
HbA1C: Glycated hemoglobin
HDI: Human Development Index
HDL: High-density lipoprotein cholesterol
IPAQ: International Physical Activity Questionnaire
JIS: Joint Interim Statement
MetS: Metabolic syndrome
NC: Neck circumference
NCDs: Chronic non-communicable diseases
OR: Odds ratio
PAL: Physical activity level
POF: Household Budget Survey
PR: Prevalence ratio
Q1: 1^st^ quarter
Q2: 2^nd^ quarter
Q3: 3^rd^ quarter
Q4: 4^th^ quarter
R24h: 24-hour dietary recall
TG: Triglycerides
UPF: Ultra-processed food
VIGITEL: Surveillance system of risk and protective factors for chronic diseases by telephone survey
WC: Waist circumference
WHtR: Waist-to-height ratio
