# Supplementary material for: Ultra-processed food consumption and metabolic syndrome: a cross-sectional study in Quilombola communities of Alagoas, Brazil
Source: Int J Equity Health. 2023 Jan 17;22:14. doi: 10.1186/s12939-022-01816-z (PMC9847020; doi:10.1186/s12939-022-01816-z)
Supplement: Supplementary file 1 — Additional file 1: Supplementary Table 1. Distribution of the metabolic syndrome according to the categories of its components in quilombola women from Alagoas, Brazil, 2018. Supplementary Table 2. Mean total energy intake and absolute and relative contribution of this intake that was derived from ultra-processed foods. Supplementary Table 3. Percent distribution of the consumption, on the day before the interview, of food groups included in the Nova score of consumption of ultra-processed foods by Quilombola women in Alagoas, Brazil, 2018. Supplementary Tables 4. Description of the covariates (confounding factors) controlled in the adjusted analysis of table 4, according to the outcome evaluated. Supplementary Tables 5. Description of the covariates (confounding factors) controlled in the adjusted analysis of Table 5, according to the outcome evaluated. [file 12939_2022_1816_MOESM1_ESM.docx]

**SUPPLEMENTARY TABLES**

**Supplementary Table 1** – Distribution of the metabolic syndrome according to the categories of its components in quilombola women from Alagoas, Brazil, 2018.

| **Variables** | | **Total (n=895)**  **n (%)** | **Metabolic syndrome** | |
| --- | --- | --- | --- | --- |
|  |  |  | **Absence**  **(n = 463; 51.7%)**  **n (%)** | **Presence**  **(n = 432; 48.3%)**  **n (%)** |
| **Abdominal obesity** | |  |  |  |
|  | No | 281 (31.8) | 264 (94.0) | 17 (6.0) |
|  | Yes | 602 (68.2) | 192 (31.9) | 410 (68.1) |
| **Arterial hypertension** | |  |  |  |
|  | No | 487 (54.9) | 369 (75.8) | 118 (24.2) |
|  | Yes | 399 (45.0) | 90 (22.6) | 309 (77.4) |
| **Diabetes mellitus** | |  |  |  |
|  | No | 611 (71.2) | 416 (68.1) | 195 (31.9) |
|  | Yes | 247 (28.8) | 33 (13.4) | 214 (86.6) |
| **Hypertriglyceridemia** | |  |  |  |
|  | No | 591 (66.9) | 436 (73.8) | 155 (26.2) |
|  | Yes | 292 (33.1) | 27 (9.3) | 265 (90.7) |
| **HDL low** | |  |  |  |
|  | No | 221 (25.1) | 191 (86.4) | 30 (13.6) |
|  | Yes | 660 (74.9) | 272 (41.2) | 388 (58.8) |

**Supplementary Table 2** – Mean total energy intake and absolute and relative contribution of this intake that was derived from ultra-processed foods.

| **Quartiles of percentage contribution of caloric intake of ultra-processed foods in relation to total energy consumption** | **Total calories consumed** | **Calories consumed from ultra-processed foods** | **Contribution of caloric intake from processed foods in relation to total caloric intake** |
| --- | --- | --- | --- |
| Q1 | 1,304.9 | 0.0 | 0.0% |
| Q2 | 1,581.8 | 86.6 | 5,7% |
| Q3 | 1,427.0 | 251.4 | 17,7% |
| Q4 | 1,438.6 | 583.0 | 40,5% |

Q1= 1^st^ quarter; Q2 = 2 ^nd^ quarter; Q3 = 3^rd^ quarter; Q4 = 4^th^ quarter

**Supplementary Table 3** - Percent distribution of the consumption, on the day before the interview, of food groups included in the Nova score of consumption of ultra-processed foods by Quilombola women in Alagoas, Brazil, 2018.

| **Ultra-processed food groups** | **n (%)** |
| --- | --- |
| - Packaged snacks (or chips) or saltine crackers | 233 (26.0) |
| - Margarine | 181 (20.2) |
| - Sweet biscuits with or without filling | 148 (16.5) |
| - Powdered drink mix (Tang type) | 130 (14.5) |
| - Loaves of bread, like of the hot dogs or hamburgers buns or similar | 122 (13.6) |
| - Regular or diet soda | 94 (10.5) |
| - Ready-made sauce, seasonings, and industrialized broths | 49 (5.5) |
| - Pizza, savory snacks (patty, deep chicken-filled fried croquette, croissant), pastry, or industrialized popcorn | 46 (5.1) |
| - Sausage, hamburger or nuggets | 44 (4.9) |
| - Ham, salami or mortadella | 36 (4.0) |
| - Package cake, stuffed cake, stuffed cake with icing, cupcake, sweet pies, pudding, or churros | 24 (2.7) |
| - Chocolate in bars, chocolate truffle, industrialized sweets, chewing gum, candies, lollipops, mousse, peanut candy, or condensed milk | 17 (1.9) |
| - Ice cream or popsicle | 13 (1.4) |
| - Mayonnaise, ketchup, mustard, or cream curd | 12 (1.3) |
| - Instant noodles (Miojo-type) or packaged soup | 10 (1.1) |
| - Chocolate drink (Nescau-type) or shake drink | 8 (0.89) |
| - Lasagna | 7 (0.78) |
| - Canned or bottled fruit juice (Del Valle-type) | 6 (0.67) |
| - Breakfast cereal (Sucrilhos-type) | 3 (0.34) |
| - Fruit- or chocolate-flavored yogurt | 3 (0.34) |
| - French fries/potato straw | 1 (0.11) |

**Supplementary Tables 4 -** Description of the covariates (confounding factors) controlled in the adjusted analysis of table 4, according to the outcome evaluated.

| **Outcomes** | **Model 1** | **Model 2** | **Model 3 *** |
| --- | --- | --- | --- |
| Hyperglycemia | Adjusted for demographic and socioeconomic characteristics, which crude analysis showed p≤ 0.20 (age, race/skin color, marital status, schooling, family participation in a government program, family income, employment status, and food insecurity). | Adjusted for lifestyle and health variables that the crude analysis showed p≤ 0.20 (smoking and health problems in the last 15 days) added to the variables of model 1 that showed p <0.05 (age, education, race and food insecurity) | Adjusted for anthropometric variables that the crude analysis showed p ≤0.20 (excess weight and neck circumference) added to the model 1 variables that showed p <0.05 (age, education, race and food insecurity). |
| Systemic arterial hypertension | Idem | Idem, added to the variables of model 1 that showed p <0.05 (age and employment status). | Idem, added to the model 1 variables that showed p <0.05 (age and employment status). |
| Abdominal obesity | Idem | Idem, added to the variables of model 1 that showed p <0.05 (age, marital status and food insecurity). | Idem, added to the model 1 variables that showed p <0.05 (age, marital status and food insecurity) |
| Low HDL | Idem | Idem, added to the variables of model 1 that showed p <0.05 (marital status and participation in government programs). | Idem, added to the model 1 variables that showed p <0.05 (marital status and participation in government programs). |
| Hypertriglyceridemia | Idem | Idem, added to the variables of model 1 that showed p <0.05 (age). | Idem, added to the model 1 variables that showed p <0.05 (age). |
| Metabolic syndrome | Idem | Idem, added to the variables of model 1 that showed p <0.05 (age, employment status and family income). | Idem, added to the model 1 variables that showed p <0.05 (age, employment status and family income). |

* Lifestyle and health variables were not part of this final model as they did not present p < 0.05 in the analysis of model 2.

**Supplementary Tables 5 -** Description of the covariates (confounding factors) controlled in the adjusted analysis of Table 5, according to the outcome evaluated.

| **Outcomes** | **Model 1** | **Model 2** | **Model 3 *** |
| --- | --- | --- | --- |
| Hyperglycemia | Adjusted for demographic and socioeconomic characteristics, which crude analysis showed p≤ 0.20 (age, race/skin color, marital status, schooling, family participation in a government program, family income, employment status, and food insecurity | Adjusted for lifestyle and health variables that the crude analysis showed p≤ 0.20 (smoking and health problems in the last 15 days) added to the variables of model 1 that showed p <0.05 (age, education and race) | Adjusted for anthropometric variables that the crude analysis showed p ≤0.20 (excess weight and neck circumference) added to the model 1 variables that showed p <0.05 (age, education and race and food insecurity). |
| Systemic arterial hypertension | Idem | Idem, added to the variables of model 1 that showed p <0.05 (age, (employment status and family income). | Idem, added to the model 1 variables that showed p <0.05 (age, employment status and family income). |
| Abdominal obesity | Idem | Idem, added to the variables of model 1 that showed p <0.05 (age, marital status and insecurity food) | Idem, added to the model 1 variables that showed p <0.05 (age, marital status and insecurity food) |
| Low HDL | Idem | Adjusted for lifestyle and health variables that the crude analysis showed p≤ 0.20 (smoking and health problems in the last 15 days) ** | Adjusted for anthropometric variables that the crude analysis showed p ≤0.20 (excess weight and neck circumference) ** |
| Hypertriglyceridemia | Idem | Idem, added to the variables of model 1 that showed p <0.05 (age). | Idem, (age). |
| Metabolic syndrome | Idem | Idem, added to the variables of model 1 that showed p <0.05 (age and family income). | Idem, (age and family income) |

* Lifestyle and health variables were not part of this final model as they did not present p < 0.05 in the analysis of model 2.

** None of the Model 1 variables presented p <0.05. Thus, none of them were added to models 2 and 3.
